# Cardiovascular Magnetic Resonance in Patients with Cardiac Electronic Devices: Evidence from a Multicenter Study

**DOI:** 10.3390/jcm12206673

**Published:** 2023-10-22

**Authors:** Andrea Barison, Fabrizio Ricci, Anna Giulia Pavon, Giuseppe Muscogiuri, Giandomenico Bisaccia, Giovanni Camastra, Manuel De Lazzari, Chiara Lanzillo, Mario Raguso, Lorenzo Monti, Sara Vargiu, Patrizia Pedrotti, Marcello Piacenti, Giancarlo Todiere, Gianluca Pontone, Ciro Indolfi, Santo Dellegrottaglie, Massimo Lombardi, Juerg Schwitter, Giovanni Donato Aquaro, on behalf of the RICAMI Investigators (Risonanza Magnetica Cardiaca nei Portatori di PM/ICD), on behalf of the Working Group on Cardiovascular Magnetic Resonance of the Italian Society of Cardiology

**Affiliations:** 1 Fondazione Toscana Gabriele Monasterio, 56127 Pisa, Italy; 2 Life Science Institute, Scuola Superiore Sant’Anna, 56127 Pisa, Italy; 3 Department of Neuroscience, Imaging and Clinical Sciences, G. d’Annunzio University of Chieti-Pescara, 66100 Chieti, Italy; 4 Cardiocentro Ticino Institute, Ente Ospedaliero Cantonale, 6900 Lugano, Switzerland; 5 Department of Perioperative Cardiology and Cardiovascular Imaging, Centro Cardiologico Monzino IRCCS, 20138 Milan, Italy; 6 Ospedale MG Vannini, 00177 Roma, Italy; 7 Department of Cardiac Thoracic and Vascular Sciences and Public Health, University of Padua, 35122 Padova, Italy; 8 Ospedale Policlinico Casilino, 00169 Roma, Italy; 9 IRCCS Humanitas Research Hospital, 20089 Rozzano, Italy; 10 Cardiologia 3, ASST Grande Ospedale Metropolitano Niguarda, 20162 Milan, Italy; 11 Cardiac Magnetic Resonance Laboratory, Cardiologia 4, ASST Grande Ospedale Metropolitano Niguarda, 20162 Milan, Italy; 12 Department of Biomedical, Surgical and Dental Sciences, University of Milan, 20122 Milan, Italy; 13 Division of Cardiology, Magna Graecia University, 88100 Catanzaro, Italy; 14 Center for Cardiovascular Research, Magna Graecia University, 88100 Catanzaro, Italy; 15 Mediterranea Cardiocentro, 80122 Naples, Italy; 16 Advanced Cardiovascular Imaging Unit, Ospedale Medico-Chirurgico Accreditato Villa dei Fiori, 80011 Acerra, Italy; 17 Multimodality Cardiac Imaging Section, IRCCS Policlinico San Donato, 20097 Milan, Italy; 18 Division of Cardiology, Cardiovascular Department, University Hospital Lausanne—CHUV, 1011 Lausanne, Switzerland; 19 CMR Center, University Hospital Lausanne—CHUV, 1011 Lausanne, Switzerland; 20 Faculty of Biology & Medicine, University of Lausanne—UniL, 1015 Lausanne, Switzerland; 21 Academic Radiology Unit, Department of Surgical Medical and Molecular Pathology and Critical Area, University of Pisa, 56126 Pisa, Italy

**Keywords:** cardiovascular magnetic resonance, safety, cardiac electronic devices, pacemaker, defibrillator

## Abstract

Background: Most recent cardiac implantable electronic devices (CIEDs) can safely undergo a cardiovascular magnetic resonance (CMR) scan under certain conditions, but metal artifacts may degrade image quality. The aim of this study was to assess the overall diagnostic yield of CMR and the extent of metal artifacts in a multicenter, multivendor study on CIED patients referred for CMR. Methods: We analyzed 309 CMR scans from 292 patients (age 57 ± 16 years, 219 male) with an MR-conditional pacemaker (*n* = 122), defibrillator (n = 149), or loop recorder (n = 38); CMR scans were performed in 10 centers from 2012 to 2020; MR-unsafe implants were excluded. Clinical and device parameters were recorded before and after the CMR scan. A visual analysis of metal artifacts was performed for each sequence on a segmental basis, based on a 5-point artifact score. Results: The vast majority of CMR scans (n = 255, 83%) were completely performed, while only 32 (10%) were interrupted soon after the first sequences and 22 (7%) were only partly acquired; CMR quality was non-diagnostic in 34 (11%) scans, poor (<1/3 sequences were diagnostic) in 25 (8%), or acceptable (1/3 to 2/3 sequences were diagnostic) in 40 (13%), while most scans (n = 201, 68%) were of overall good quality. No adverse event or device malfunctioning occurred, and only nonsignificant changes in device parameters were recorded. The most affected sequences were SSFP (median score 0.32 [interquartile range 0.07–0.91]), followed by GRE (0.18 [0.02–0.59]) and LGE (0.14 [0.02–0.55]). ICDs induced more artifacts (median score in SSFP images 0.87 [0.50–1.46]) than PMs (0.11 [0.03–0.28]) or ILRs (0.11 [0.00–0.56]). Moreover, most artifacts were located in the anterior, anteroseptal, anterolateral, and apical segments of the LV and in the outflow tract of the RV. Conclusions: CMR is a versatile imaging technique, with a high safety profile and overall good image quality even in patients with MR-conditional CIEDs. Several strategies are now available to optimize image quality, substantially enhancing overall diagnostic yield.

## 1. Introduction

Cardiovascular magnetic resonance (CMR) is a multiparametric, highly reproducible, and comprehensive imaging technique, with a wide range of clinical applications [1]. Overall, it is highly appreciated and prescribed for its safety, feasibility, and non-invasiveness, although the presence of any ferromagnetic, conductive, or electronic material should be carefully screened and checked for possible interactions with CMR magnetic fields [2,3,4,5,6].

Patients with cardiac implantable electronic devices (CIEDs) represent a non-negligible proportion of patients referred for CMR, including pacemakers (PMs), implantable cardioverter defibrillators (ICDs), and implantable loop recorders (ILRs). Their metal and electronic components are subject to mechanical, electrical, and heating effects induced by CMR magnetic fields, with possible harmful risks for patients [7,8]. To minimize these concerns, major efforts have been made to design CIEDs’ hardware and software to make the devices suitable for CMR. Currently, most CIEDs are MR-conditional, i.e., they pose no hazards in a specified MR environment and under pre-specified conditions stated by the implant manufacturer (i.e., field strength, slew rate, SAR, device position, patient temperature). Before the scan, the MR scan mode should be activated; during the scan, all specific CIED scanning conditions (SAR, gradient slew rate, etc.) should be respected; after the scan, the previous PM/ICD programming should be reactivated [6,9]. The presence of older implants and abandoned, fractured, or epicardial leads make the system MR-unsafe, and, unless deemed clinically mandatory, the CMR should be replaced by other imaging modalities. Patients with ILR can be safely scanned soon after implantation, without any special programming; all stored data should be downloaded before CMR because of the risk of data loss; moreover, artifactual arrhythmic events might be recorded during CMR [10].

Another limitation of CMR in CIED patients is the generation of metal artifacts, which can degrade image quality, with a variable extent depending on several factors, including the size, shape, type of metal, and the physical location of the pulse generator. In 2011, Sasaki et al. [11] evaluated 71 CMR studies in patients with PMs or ICDs: in contrast to patients with a right-sided PM/ICD and left-sided PM, patients with a left-sided ICD presented a higher burden of artifacts, particularly in the anterior and apical left ventricular (LV) segments. In 150 patients with an MR-conditional PM from a single vendor, cine steady-state free precession (SSFP) images were of good to excellent quality in most cases, and only 5% of the LV and 2% of the right ventricular acquisitions were non-diagnostic [12]. In another study on 72 CMR scans performed in CIED patients, SSFP cine imaging resulted in a higher rate of non-diagnostic imaging (22%) than cine spoiled gradient echo (GRE) (1%) and late gadolinium enhancement (LGE) sequences (2%), but CMR provided diagnostic or management-changing information in the majority (63%) of patients [13]. In a study on 128 CIED patients, generator type and location were highly influential with regard to CMR image quality [14]: cine SSFP imaging was found to be mostly non-diagnostic in ICD patients, but a significant improvement in image quality was demonstrated with GRE imaging, in particular when acquired after contrast injection. LGE was non-diagnostic in about one-third of LV segments of ICD patients but was artifact-free in >94% segments for all other device types. Similarly, in a study on 120 patients with CIEDs, cine SSFP imaging of the right ventricle (RV) was non-diagnostic in patients with ICDs and ILRs, while GRE sequences displayed fewer artifacts [15]. Overall, all these studies demonstrated that ICDs and subcutaneous ICDs produce higher image distortion than PMs because of their larger size. Right-sided PMs and ILRs produce less artifacts than conventional left-sided PMs and ICDs. Pacing and defibrillating leads cause only minor artifacts [16]. Steady-state free precession (SSFP) cine sequences generate more artifacts than fast spin echo (FSE) and spoiled gradient echo (GRE) sequences, so that the latter can be used for cardiac cine imaging. Wideband LGE sequences have been developed to minimize metal artifacts [17,18,19].

Currently, the imaging of CIED patients with MR scanners is not yet readily available in all centers, being primarily limited to advanced imaging laboratories requiring skilled technicians and a dedicated cardiological service to perform CIED programming before and after each CMR scan. The aim of this study was to assess the overall diagnostic yield of CMR and the extent and clinical implications of metal artifacts in a multicenter, multivendor study on CIED patients referred for CMR.

## 2. Materials and Methods

### 2.1. Subjects

We prospectively enrolled all patients referred for CMR in 10 Centers between 2012 and 2020 with any MR-conditional CIED (see Supplementary Table S1 for a detailed list of the participating centers). Patients with MR-unsafe implants were excluded, as were patients in which the study was not performed or included only localizer images. The study protocol had been approved by the Local Ethical Committee, and all patients provided written informed consent before enrolment. Clinical and device parameters were recorded before and after the CMR scan by an experienced electrophysiologist. In particular, battery status and sensing/pacing thresholds of all leads were documented, and the device memory was evaluated for events (e.g., appropriately or inappropriately classified arrhythmias). All devices were programmed into the MR-safe mode strictly following the recommendations of the manufacturer, either to sensing-only (ODO or OVO) or to asynchronous pacing (VOO), depending on the patient’s intrinsic rhythm. All tachyarrhythmia functions (monitoring, anti-tachycardia pacing, and defibrillation) were turned off before CMR. Immediately after the CMR scan, all devices underwent complete examination and were reprogrammed to their original settings.

### 2.2. CMR Imaging

Nearly all exams (n = 305) were performed on 1.5 T MR scanners (169 General Electric, 22 Philips, 114 Siemens), while only 3 exams were performed on a 3T scanner (Philips, Ingenia). CMR scanning protocols were tailored to the clinical indication, with particular care to comply with device manufacturers’ recommendations about slew rate and specific absorption rate. Patients were continuously monitored throughout the entire scan by an experienced cardiologist, using vector–surface ECG, peripheral pulse oximetry, respiratory motion pattern, and non-invasive blood pressure measurements. All efforts were made to limit metal artifacts and to acquire as many diagnostic images as possible, according to the SCMR recommendation on scanning protocols [1]. For the assessment of cardiac anatomy and function, SSFP cine imaging was typically used; in patients with non-diagnostic image quality, SSFP imaging was subsequently replaced with a GRE cine sequence. According to clinical indication, in some patients, black blood images were also acquired, using T1, T2, or proton density-weighted FSE sequences. Gadolinium-based contrast agents were administered (0.1–0.2 mmol/kg bodyweight) for perfusion, angiographic, or late enhancement (LGE) imaging. The perfusion scan and late enhancement imaging were performed using GRE sequences; for LGE imaging, the inversion–recovery pre-pulse delay was determined from an inversion-prepared cine scan (Look-Locker) and individually adjusted to optimally suppress the signal from normal myocardium; when available, wideband LGE sequences were preferred to reduce artifacts. In some patients, single-shot SSFP sequences for perfusion and/or LGE were also acquired but were excluded from the analysis.

### 2.3. Image Quality Assessment

A visual analysis of metal artifacts was performed for each sequence on a segmental basis by experienced readers (>10 years of experience in CMR). First, a general 4-point grading of the quality and diagnostic yield of each CMR scan was provided, relative to the clinical indication of each exam: non-diagnostic (i.e., there were no interpretable sequences), poor (<1/3 of the initially programmed sequences were interpretable), acceptable (1/3 to 2/3 of the initially programmed sequences were interpretable), or good (>2/3 of the initially programmed sequences were interpretable). This first quality score was assessed taking into account the clinical indication and the number of CMR sequences theoretically needed to address it, according to the 2020 SCMR recommendation on scanning protocols as a reference standard [1]. Moreover, for each CMR scan, it was evaluated whether it provided new (i.e., management-changing) diagnostic information that had not been provided by any other imaging or clinical data.

**Figure 1:**
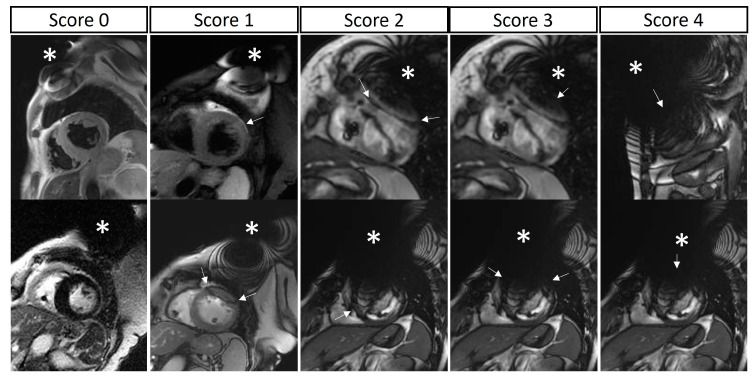
Segmental artifact grading. A 5-point grading of lead- or generator-related artifacts was calculated for each myocardial segment. The two rows show two example cases for each score (from **left** to **right**): 0, no artifacts; 1, mild artifact (obscuring <1/3 of the segment); 2, moderate artifact (obscuring 1/3 to 2/3 of the segment); 3, severe artifact (obscuring >2/3 of the segment); and 4, complete artifact (the segment is completely obscured by artifacts). Device generators are indicated with an asterisk; artifacts are indicated with arrows; please note that, in the same image, different segments may present different degrees of artifacts.

Second, a detailed 5-point grading of lead- or generator-related artifacts was provided for each myocardial segment for each acquired sequence: 0, no artifacts; 1, mild artifact (obscuring <1/3 of the segment); 2, moderate artifact (obscuring 1/3 to 2/3 of the segment); 3, severe artifact (obscuring >2/3 of the segment); or 4, complete artifact (the segment was completely obscured by artifacts) (Figure 1). The LV was segmented according to the standard 17-segment model, while the RV was segmented into 4 macro-areas (basal free wall, mid free wall, apex, outflow tract) (Supplementary Figure S1). The atria were segmented into 5 macro-areas (interatrial septum, right atrial roof, right atrial lateral wall, left atrial roof, left atrial lateral wall) (Supplementary Figure S2). Segmental analysis of LV and RV artifacts was performed in both short- and long-axis views, taking into account all acquired sequences, while segmental analysis of left and right atrial artifacts was performed only from long-axis views (because the atria were generally not included in short-axis acquisitions). For each patient, a sequence-specific global biventricular artifact score was calculated as an average of all LV and RV segmental scores from both the short- and long-axis views of the same sequence type. Similarly, for each patient, a sequence-specific global biatrial artifact score was calculated as an average of all left and right atrial walls, including the interatrial septum, from the long-axis views of the same sequence type.

**Figure 2:**
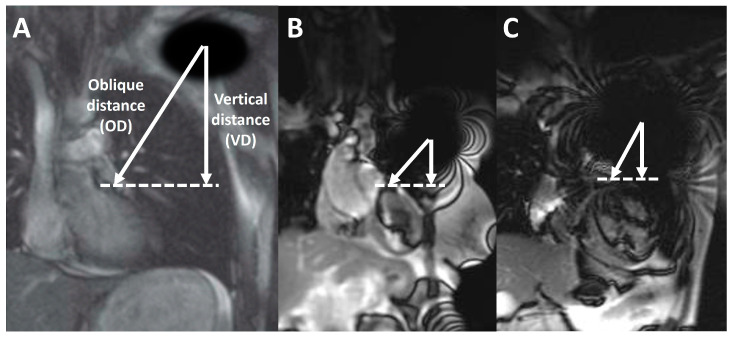
Device-to-heart distance, calculated on coronal scout images as the vertical distance (VD, vertical arrow) and as the oblique distance (OD, oblique arrow) estimated from the center of the generator artifact (center of the signal void in the left shoulder, asterisk) to the anterior interventricular septum (dashed line). Alongside, a schematic picture (**A**) and the measurements from a patient with pacemaker (**B**) and a patient with a defibrillator (**C**) are represented.

In addition, in coronal scout imaging, the vertical and the oblique distances between the center of the generator-related signal void to the anterior basal septum were measured (Figure 2); a negative distance was considered for devices (such as subcutaneous ICDs) projecting over the heart in coronal scout images.

### 2.4. Statistical Analysis

All data were analyzed using SPSS version 20.0 (IBM Corp., Armonk, NY, USA) and R (https://www.r-project.org/, accessed on 10 July 2023) statistical packages. Continuous variables were described as mean ± standard deviation (SD) if normally distributed, while as median (interquartile range) if not normally distributed; categorical variables were described as frequencies and percentages. In particular, all segmental artifact scores were calculated as the median (interquartile range) values of all patients for each segment, distinguished according to device and sequence type. Comparison between groups was conducted using the independent-samples *t*-test for continuous values with normal distribution, while the Wilcoxon rank-sum test was applied for continuous values with a non-normal distribution. The χ2 testing was performed for non-continuous variables. Correlation analysis was performed using Pearson’s test or Spearman’s test where appropriate. A linear regression analysis was used to determine the effects of several variables on the number and severity of artifacts. A 2-tailed *p* < 0.05 was considered statistically significant.

## 3. Results

We analyzed 309 CMR scans from 292 patients (age 57 ± 16 years, 219 male); the most common underlying etiology was non-ischemic heart disease (Table 1; Supplementary Table S2). MR-conditional devices included pacemakers (n = 122), defibrillators (n = 149), and loop recorders (n = 38) (Supplementary Table S3). All implants were in the left hemithorax, except for two PMs, three ICDs, and one ILR. Nearly all scans included cine SSFP and late enhancement imaging, while other sequences (GRE, BB (black blood)-FSE, STIR (short tau inversion recovery)-FSE, perfusion) were performed in one-fourth to one-third of cases according to the clinical indication (Table 2).

The vast majority of CMR scans (n = 255, 83%) were completely performed, while only 32 (10%) were interrupted soon after the first sequences, and 22 (7%) were only partly acquired. CMR quality was non-diagnostic in 34 (11%) scans (including all the 32 scans that had been interrupted after the first sequences), poor (<1/3 of the initially programmed sequences were diagnostic) in 25 (8%), or acceptable (1/3 to 2/3 of the initially programmed sequences were diagnostic) in 40 (13%), while most scans (n = 210, 68%) were of overall good quality (Table 3). CMR provided a new (i.e., management-changing) diagnosis in 119 (39%) cases.

**Figure 3:**
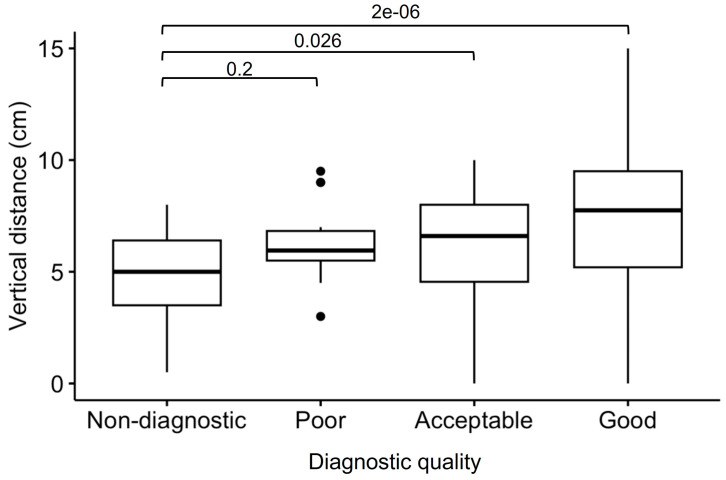
Generator-to-heart vertical distance, distinguished between CMR scans with different image quality. Outliers are indicated with dots.

The estimated vertical distance between the center of the generator-related signal void to the anterior basal septum on coronal scout imaging was significantly different among patients with different image quality (Figure 3) and was shown to be a significant predictor of overall scan quality through univariate regression analysis (OR 1.12, 95% confidence interval 1.04–1.20, *p* = 0.0004). Moreover, ICDs were more frequently associated with a worse scan quality, compared to PMs and ILRs (Table 4).

**Figure 4:**
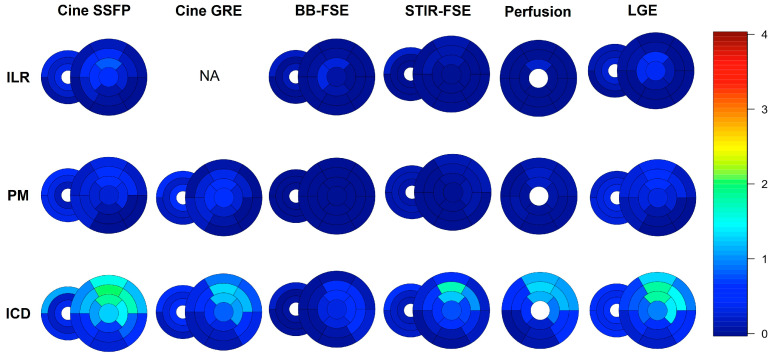
Bull’s eye of segmental artifacts across different CMR sequences and different devices, from short-axis images; the median score of each segment is represented with different colors. Please see Supplemental Methods for further details.

**Figure 5:**
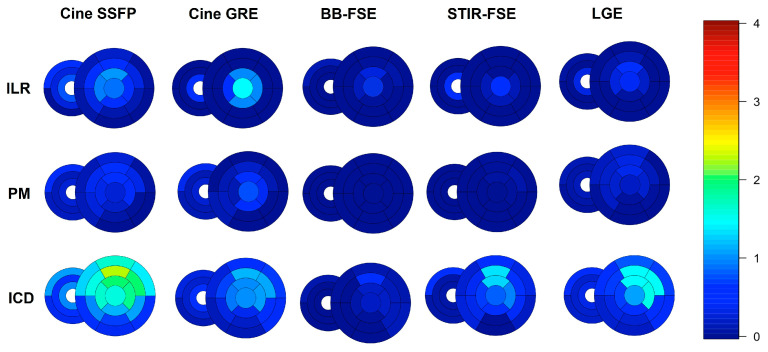
Bull’s eye of segmental artifacts across different CMR sequences and different devices, from long-axis images; the median score of each segment is represented with different colors. Please see Supplemental Methods for further details.

A detailed segmental analysis of metal artifacts across the different CMR sequences and the different devices is presented in Figure 4 and Figure 5. Global biventricular artifact scores across the different CMR sequences are presented in Supplementary Table S4. In particular, the most affected sequences were SSFP (median global biventricular score 0.32 [interquartile range 0.07–0.91]), followed by GRE (0.18 [0.02–0.59]) and LGE (0.14 [0.02–0.55]). ICDs induced more artifacts (median global biventricular score in SSFP images 0.87 [0.50–1.46]) than PMs (0.11 [0.03–0.28]) or ILRs (0.11 [0.00–0.56]); subcutaneous ICDs (n = 15) produced the highest rate of artifacts among ICD subtypes (median score in SSFP images 1.36 [1.08–1.86]). In patients with leadless PMs (n = 2), metal artifacts were limited to the right ventricular free wall and the mid interventricular septum and were more apparent in cine SSFP images. Right-sided PMs (n = 2) and ILR (n = 1) did not produce any artifacts, while right-sided ICDs (n = 3) produced only mild-to-moderate artifacts limited to the RV and right atrium. Most artifacts were located in the anterior (segments 1,7: median score in SSFP images 0.75 [0.00–2.00]), anteroseptal (segments 2,8: median score in SSFP images 0.25 [0.00–1.50]), anterolateral (segments 6,12: median score in SSFP images 0.50 [0.00–1.50]), and apical segments (segments 13 to 17: median score in SSFP images 0.50 [0.00–1.50]) of the LV.

The prevalence of metal artifacts affecting either the right or left atrium was very low, as apparent from the fact that almost all global biatrial artifact scores were close to zero (Supplementary Table S5). In particular, the very few artifacts were limited to the right atrial roof, usually related to the proximity of the right atrial pacing lead, and in almost all cases, they did not significantly degrade image quality.

No adverse event or device malfunctioning occurred, and only nonsignificant changes in device parameters were recorded (Supplementary Table S6).

## 4. Discussion

In this multicenter study, all patients carrying an MR-conditional device underwent a safe CMR scan without experiencing any device malfunctioning, and the extent of metal artifacts made the CMR non-diagnostic only in a minority (11%) of cases. Overall, the most affected sequences were SSFP, followed by GRE and LGE; ICDs induced larger artifacts than PMs or ILRs. Most artifacts were observed in the anterior, anteroseptal, anterolateral, and apical segments of the LV and in the outflow tract of the RV. The proximity of the CIED to the heart directly correlated with the extent of artifacts observed on CMR images, with closer device position leading to more pronounced artifact formation.

Our results, derived from a large multicenter cohort, reinforce and expand upon prior evidence [11,12,13,14,15], confirming the feasibility and reliability of CMR imaging in CIED recipients, with the inherent limitations of metal artifacts according to device type, location, and CMR sequences. In particular, our study is among the largest published so far to provide an overall assessment of the feasibility and diagnostic yield of CMR in CIED patients, as well as a detailed analysis of artifact severity and location across different CMR sequences and device types. Both large-volume and small-volume CMR centers with different equipment, logistics, and geographic location were included in this multicenter study, providing a real-life picture of the strengths and difficulties of performing CMR exams in patients with electronic devices. Our results demonstrate a high proportion of diagnostic quality, i.e., in 98% of PM patients and in approximately 85% of ICD and ILR patients, which favors a CMR exam in all device patients, including ICD and ILR patients. Similarly to a previous single-center study on 72 CIED patients, where CMR provided diagnostic or management-changing information in the majority (63%) of patients [13], in our study, 68% of scans were of overall good diagnostic quality and 39% provided management-changing information.

As far as the segmental analysis of metal artifacts is concerned, our results are in line with a previous study on 128 CIED patients, where generator type and location were highly influential with regard to CMR image quality [14]. The authors of that study proposed an algorithm, whereby all conventionally employed CMR sequences may be used in most ILRs and right-sided PMs, and GRE sequences should be used instead of SSFP modules in ICDs, while an initial trial using SSFP cine imaging may be used in left-sided PMs with the recommendation to switch to GRE modules (better if acquired after contrast injection) in cases where an impaired image quality is found. In the same study, all patients underwent a routine chest X-ray prior to the CMR examination in order to measure the minimal distance between the inferior border of the device and the heart silhouette in the anterior–posterior projection: this generator–heart distance was correlated with the artifact burden, similarly to the approximate vertical distance we calculated between the center of the generator-related signal void to the anterior basal septum on coronal scout imaging, which we also found to be a significant predictor of overall scan quality. In another study on 57 CIED patients, a CMR scoring system aimed at predicting the extent of metal artifacts and CMR image quality has been proposed, based on simple and readily available information gathered from chest radiography and vendor-specific features of the CIED [20]. Notably, a distance shorter than 11.65 cm from the generator to the LV apex and an RV lead diameter exceeding 6.45 Fr emerged as independent predictors of CMR artifacts: these parameters were integrated into an artifact prediction score (i.e., DR-CAPS) to enhance the optimization of patient selection and to improve the overall quality of CMR imaging.

Moreover, our findings support the use of perfusion imaging with CMR in CIED patients: a rest perfusion module was indeed acquired in nearly a third of scans and showed fewer artifacts than cine and LGE imaging, limited almost exclusively to ICD patients. On the other hand, only eight stress perfusion scans were acquired in our cohort. In a study on 66 patients with MR-conditional CIEDs, stress CMR was safely performed using a GRE perfusion module (50 patients under continuous pacing) and was diagnostic in 98% of cases [21]. Similar results were also found in 20 patients undergoing regadenoson stress CMR [22]. Nevertheless, several other studies are needed to investigate the feasibility and costs of stress CMR compared to other more widespread stress imaging modalities in CIED patients.

In our study, only two patients with a leadless PM were included: in both cases, metal artifacts were limited to the right ventricular free wall and the mid interventricular septum and were more apparent in cine SSFP images. Our findings seem to be in line with a recent study on 15 patients with leadless PMs, where CMR imaging showed good image quality without any clinical or device-related adverse events: artifacts were mostly confined to the mid and apical RV free wall, to the mid and apical interventricular septum, and to the LV apex, while the quantification of LV function was feasible in all patients [23].

Currently, several approaches have been developed to reduce metal artifacts. The importance of wideband LGE was confirmed in a study on 133 patients, in whom it changed clinical management in an additional 39 (75%) ICD patients and 10 (19%) PM patients when compared to conventional LGE imaging [19]. Right-sided PM implantation should be considered in CIED patients requiring subsequent CMR imaging to ensure sufficient image quality. Moreover, arm-raised imaging represents a straightforward method to reduce CMR artifacts in patients with left-sided CIEDs and can be used alongside other image quality improvement methods. In a study on 171 CMR scans in patients with CIEDs [24], patients with a right-sided pacemaker showed fewer artifacts (6.2% of segments in SSFP images) than patients with a left-sided pacemaker (17.8%, *p* < 0.001). In the same study, in patients with left-sided ICDs, arm-raised imaging reduced the artifacts from 37.5% to 12.5% (*p* = 0.02). Once CMR images have been acquired, novel deep learning approaches have been developed for automatic segmentation of cardiac structures in patients with susceptibility to artifacts from CIEDs [25].

As far as safety is concerned, our findings confirm the high safety of scanning MR-conditional devices, in line with the previous literature [26,27]. More recently, pooled data on legacy (i.e., non-MR-conditional) devices also demonstrated a good safety profile [28,29]. In another study, 615 CMR exams of non-conditional CIEDs were performed, and there were no adverse events and no clinically significant changes in device parameters [30]. Moreover, CMR safety has been proven even in patients with abandoned or epicardial leads [31,32,33,34].

Our current study presents several limitations that should be acknowledged. First, it enrolled patients with a clinical indication for CMR imaging from different CMR centers, so that CMR sequences were chosen, acquired, and adapted according to the clinical suspicion and to the local equipment: even though every effort was made to limit metal artifacts and to acquire as many diagnostic images as possible, according to international recommendation on scanning protocols, imaging protocols were neither made forcefully homogeneous nor extended unnecessarily. Thus, this study includes a heterogeneous number of CMR pulse sequences because not all device patients underwent all CMR imaging sequences with the same parameters. For this reason, a detailed analysis of the impact of different sequence parameters on the final image quality was not performed. Second, only MR-conditional devices were included, so the same findings may not necessarily apply to non-MR-conditional CIEDs. Third, the assessment of the vertical distance of the device from the heart was performed on vertical scout imaging, while no other radiological images (such as plain chest radiograms) were specifically acquired.

## 5. Conclusions

CMR is a versatile imaging technique, with a high safety profile and very few absolute contraindications. Patients with MR-conditional CIEDs can be safely scanned and should not be denied a CMR exam when clinically indicated. Unfortunately, the imaging of patients with CIEDs is still not readily available in all centers because of logistical constraints, being limited to advanced centers with skilled technicians and with dedicated cardiological staff to perform pre- and post-scan CIED programming. The extent of metal artifacts causes signal degradation to a variable extent, according to the device type and sequence used, but several strategies are now available to extend CMR use and optimize image quality even in patients with CIEDs. In this multicenter, multivendor setting, current CMR techniques yield diagnostic quality in 98% of PM patients and in 83% and 84% in ICD and ILR patients, respectively.

## Figures and Tables

**Table 1:** Demographics.

Sample Size, n	309
Age, years	60 (48–70)
Male, n (%)	144 (74.6%)
BSA, Kg/m^2^	1.91 (1.71–2.04)
Hypertension, n (%)	122 (42.1%)
Dyslipidemia, n (%)	107 (34.6%)
Diabetes, n (%)	58 (20.0%)
Implant to CMR time, months	12.3 (5.7–29.4)
Etiology	
Non-ischemic heart disease, n (%)	169 (54.9%)
Conduction disorders, n (%)	53 (17.2%)
Ischemic heart disease, n (%)	76 (24.7%)
Valvular heart disease, n (%)	10 (3.2%)

BSA, body surface area; continuous variables are presented as median (interquartile range).

**Table 2:** CMR sequences acquired, distinguished according to device type.

Sequences, n (%)	Overall Scans	ICD	PM	ILR
	309 (100%)	149 (48.2%)	122 (39.5%)	38 (12.3%)
SSFP	250 (80.9%)	107 (71.8%)	106 (86.9%)	37 (97.4%)
GRE	82 (26.5%)	56 (37.6%)	24 (19.7%)	2 (5.3%)
BB-FSE	73 (23.6%)	33 (22.1%)	12 (9.8%)	28 (73.7%)
STIR-FSE	95 (30.7%)	44 (29.5%)	39 (32.0%)	12 (31.6%)
Perfusion	98 (31.7%)	48 (32.2%)	46 (37.7%)	4 (10.5%)
LGE	252 (81.6%)	114 (76.5%)	107 (87.7%)	31 (81.6%)
Scan duration, min	42 (30–50)	40 (25–50)	43 (34–50)	44 (29–56)

BB, black blood; FSE, fast spin echo; GRE, gradient echo; ICD, implantable cardioverter defibrillator; ILR, implantable loop recorder; LGE, late gadolinium enhancement; PM, pacemaker; SSFP, steady-state free precession; STIR, short tau inversion recovery.

**Table 3:** Overall diagnostic quality of CMR scans and anthropometric patients’ characteristics.

Overall Quality	Non-Diagnostic	Poor	Acceptable	Good	*p*
Number of CMR scans	34	25	40	210	
Age, years	54 (42–65)	58 (43–67)	58 (45–70)	61 (50–72)	0.107
BSA, Kg/m^2^	1.90 (1.71–20.7)	1.91 (1.73–2.06)	1.94 (1.72–2.10)	1.90 (1.71–2.04)	0.924
Vertical distance, cm	4.3 (1.6–6.2)	5.5 (3.8–6.8)	6.5 (3.1–8.0)	7.5 (4.8–9.5)	<0.001
Oblique distance, cm	7.9 (6.6–8.7)	9.2 (7.9–10.7)	9.2 (8.4–10.8)	9.3 (7.2–11.0)	0.553

BSA, body surface area; continuous variables are presented as median (interquartile range).

**Table 4:** Overall diagnostic quality of CMR scans and device type.

Overall Quality, n (%)	ICD (n = 149)	ILR (n = 38)	PM (n = 22)	*p*-Value
Non-diagnostic	25 (16.8)	6 (15.8)	3 (2.5)	<0.001
Poor	23 (15.4)	1 (2.6)	1 (0.8)	
Acceptable	32 (21.5)	1 (2.6)	7 (5.7)	
Good	69 (46.3)	30 (78.9)	111 (91.0)	

ICD, implantable cardioverter defibrillator; ILR, implantable loop recorder; PM, pacemaker.

## Data Availability

The data presented in this study are available upon reasonable request from the corresponding author. The data are not publicly available due to privacy reasons.

## Supplementary Materials

The following supporting information can be downloaded at https://www.mdpi.com/article/10.3390/jcm12206673/s1, Supplemental Methods; Figure S1: Representation of the regional segmentation of the LV and RV; Figure S2: Representation of the regional segmentation of the right atrium (RA) and left atrium (LA); Table S1: List of contributing centers; Table S2: Etiology of underlying heart disease; Table S3: Device characteristics; Table S4: Median global biventricular artifact score; Table S5: Median global biatrial artifact score; Table S6: Device parameters before and after the CMR scan.
