# Supplementary material for: Cardiovascular Magnetic Resonance in Patients with Cardiac Electronic Devices: Evidence from a Multicenter Study
Source: J Clin Med. 2023 Oct 22;12(20):6673. doi: 10.3390/jcm12206673 (PMC10607654; doi:10.3390/jcm12206673)
Supplement: Supplementary file 1 [file jcm-12-06673-s001.zip › jcm-2635474-supplementary.pdf]

Supplemental Material

Supplemental Methods

Biventricular bull's eye plots were constructed with the median of segment-level artifact ratings of all patients, distinguished according to device and sequence type. Each operator assigned a score of 0-4 to each of the 17 left ventricular segments as well as to basal, mid, and apical right ventricular walls and the RV outflow tract (RVOT), where a score of 0 indicates the total absence of artifacts, while a score of 4 represents an acquisition that is entirely filled with artifacts. The medians of these segment-level ratings were calculated across all patients. For RV representation, the RVOT was conventionally represented as the outer superior layer of the RV (see *Figure S1*).

Supplemental Figures

Figure S1. Representation of the regional segmentation of the LV and RV. Please note that the numbers in the LV represent the conventional 17-segment classification.

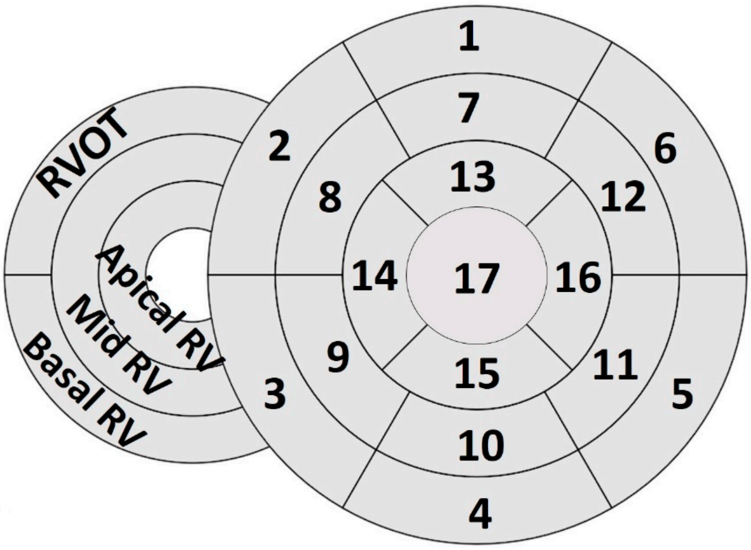

Figure S2. Representation of the regional segmentation of the right atrium (RA) and left atrium (LA). Please note that the grading of metal artifacts in the atrial walls was performed only from the long axis of each CMR sequence.

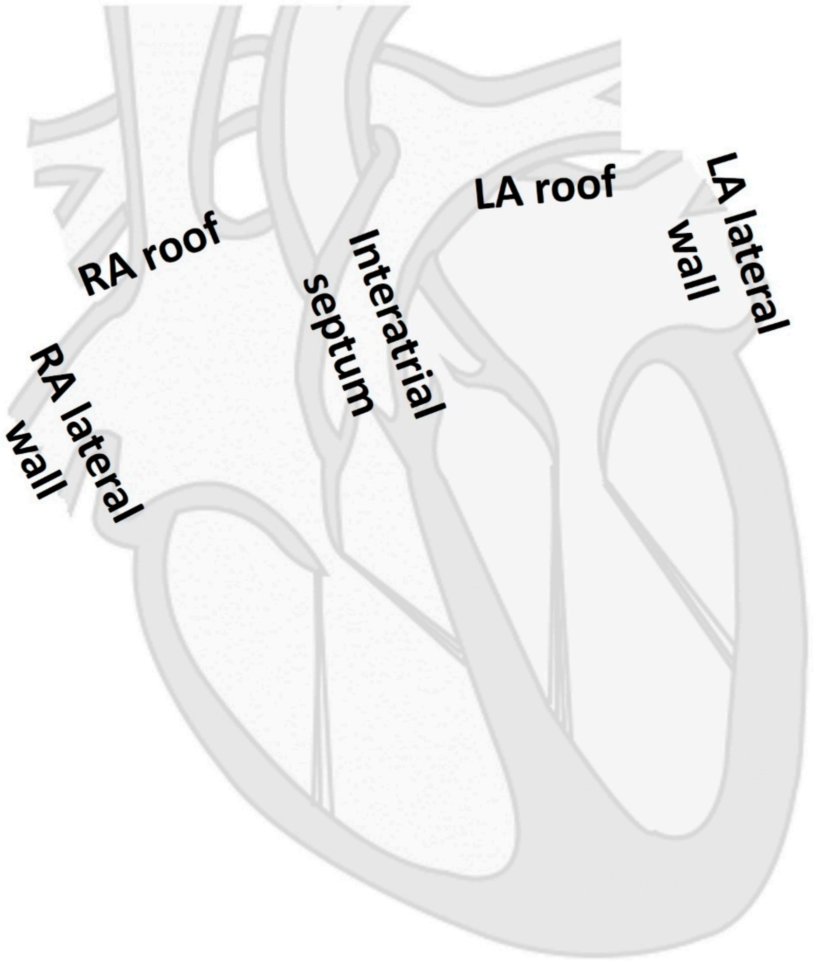

Supplemental Tables

Supplemental Table S1. List of contributing centers

**Center**	**CMR scans, n (%)**
Fondazione Toscana Gabriele Monasterio, Pisa (IT)	104 (34)
Centro Cardiologico Monzino IRCCS, Milan (IT)	68 (22)
University Hospital Lausanne, CHUV, Lausanne (CH)	48 (16)
IRCCS Policlinico San Donato, Milan (IT)	42 (14)
Ospedale Policlinico Casilino, Rome (IT)	11 (4)
ASST Grande Ospedale Metropolitano Niguarda, Milan (IT)	10 (3)
IRCCS Humanitas Research Hospital, Rozzano, Milan (IT)	10 (3)
Gabriele d'Annunzio University of Chieti-Pescara, Chieti	7 (2)
Ospedale MG Vannini, Rome (IT)	5 (2)
Department of Cardiac Thoracic and Vascular Sciences and Public Health, University of Padua (IT)	4 (1)

Supplemental Table S2. Etiology of underlying heart disease

**Etiology**	**n**	**(%)**
Ischemic heart disease	76	25
Conduction disease	51	17
Dilated cardiomyopathy	41	13
Arrhythmogenic cardiomyopathy	29	9
Hypertrophic cardiomyopathy	24	8
Pericarditis and/or myocarditis	21	7
Restrictive cardiomyopathy	17	6
Adult congenital heart disease	14	5
Other cardiomyopathies	13	4
Valve disease	10	3
Hemochromatosis	6	2
Cardiac mass	3	1
Channelopathy	2	1
Hypertensive heart disease	1	0

Supplemental Table S3. Device characteristics

**Device type**		**n**	**(%)**
ILR		38	12
PM	Single chamber	5	2
	Dual chamber	112	36
	Biventricular	3	1
	Leadless	2	1
ICD	Subcutaneous	15	5
	Single chamber	46	15
	Dual chamber	70	22
	Biventricular	18	6

ICD, implantable cardioverter defibrillator; ILR, implantable loop recorder; PM, pacemaker

Supplemental Table S4. Median global biventricular artifact score (calculated in each patient as an average of all LV and RV segmental scores from both the short- and long-axis views of the same sequence type) according to sequence and device type.

Sequences	Overall scans	ICD	PM	ILR
	n= 309 (100%)	n= 149 (48.2%)	n= 122 (39.5%)	n= 38 (12.3%)
SSFP	0.32 (0.07-0.91)	0.87 (0.50-1.46)	0.11 (0.03-0.28)	0.11 (0.00-0.56)
GRE	0.18 (0.02-0.59)	0.23 (0.10-0.81)	0.05 (0.00-0.26)	0.15 (0.07-0.24)
BB-FSE	0.00 (0.00-0.11)	0.05 (0.00-0.18)	0.00 (0.00-0.00)	0.00 (0.00-0.05)
STIR-FSE	0.05 (0.00-0.28)	0.25 (0.12-0.51)	0.00 (0.00-0.02)	0.00 (0.00-0.07)
Perfusion	0.02 (0.00-0.54)	0.52 (0.00-1.02)	0.00 (0.00-0.07)	0.00 (0.00-0.03)
LGE	0.14 (0.02-0.55)	0.42 (0.14-0.82)	0.08 (0.01-0.17)	0.00 (0.00-0.05)

BB, black blood; FSE, fast spin echo; GRE, gradient echo; ICD, implantable cardioverter defibrillator; ILR, implantable loop recorder; LGE, late gadolinium enhancement; PM, pacemaker; SSFP, steady-state free precession; STIR, short tau inversion recovery. Data are presented as median (interquartile range).

Supplemental Table S5. Median global biatrial artifact score (calculated in each patient as an average of all left and right atrial walls, including the interatrial septum, from the long-axis views of the same sequence type) according to sequence and device type.

Sequences	Overall scans	ICD	PM	ILR
	n= 309 (100%)	n= 149 (48.2%)	n= 122 (39.5%)	n= 38 (12.3%)
SSFP	0.00 (0.00-0.40)	0.20 (0.00-0.60)	0.00 (0.00-0.40)	0.00 (0.00-0.00)
GRE	0.10 (0.00-0.40)	0.00 (0.00-0.40)	0.20 (0.05-0.35)	0.00 (0.00-0.00)
BB-FSE	0.00 (0.00-0.00)	0.00 (0.00-0.00)	0.00 (0.00-0.00)	0.00 (0.00-0.00)
STIR-FSE	0.00 (0.00-0.00)	0.00 (0.00-0.00)	0.00 (0.00-0.00)	0.00 (0.00-0.00)
LGE	0.00 (0.00-0.20)	0.00 (0.00-0.30)	0.00 (0.00-0.25)	0.00 (0.00-0.00)

BB, black blood; FSE, fast spin echo; GRE, gradient echo; ICD, implantable cardioverter defibrillator; ILR, implantable loop recorder; LGE, late gadolinium enhancement; PM, pacemaker; SSFP, steady-state free precession; STIR, short tau inversion recovery. Data are presented as median (interquartile range).

Supplemental Table S6. Device parameters before and after the CMR scan.

	**Before CMR**	**After CMR**	**P-value**
Atrial lead			
Threshold (V)	0.74 (0.47)	0.70 (0.47)	0.565
Duration (ms)	0.41 (0.04)	0.41 (0.11)	0.985
Sensing (mV)	3.80 (2.19)	3.65 (2.29)	0.607
Impedance (Ω)	565.21 (265.26)	551.44 (264.58)	0.688
Right ventricular lead			
Threshold (V)	0.78 (0.40)	0.78 (0.39)	0.904
Duration (ms)	0.42 (0.10)	0.42 (0.12)	0.969
Sensing (mV)	12.07 (5.34)	12.46 (12.37)	0.722
Impedance (Ω)	514.50 (140.35)	506.22 (144.45)	0.598
Left ventricular lead			
Threshold (V)	1.46 (1.00)	1.27 (1.01)	0.672
Duration (ms)	0.65 (0.29)	0.58 (0.33)	0.638
Sensing (mV)	13.24 (8.05)	11.27 (8.98)	0.713
Impedance (Ω)	814.58 (201.40)	749.38 (296.40)	0.530

Values are presented as mean (SD).
